# Psychosocial correlates of HbA1c among adult Samoans without diabetes

**DOI:** 10.1371/journal.pmen.0000196

**Published:** 2025-02-28

**Authors:** Anna C. Rivara, Lacey W. Heinsberg, Jenna C. Carlson, Alysa Pomer, Take Naseri, Muagututia Seifuiva Reupena, Courtney C. Choy, Dongqing Wang, Erin E. Kershaw, Satupaitea Viali, Stephen T. McGarvey, Nicola L. Hawley

**Affiliations:** 1 Department of Chronic Disease Epidemiology, Yale School of Public Health, New Haven, Connecticut, United States of America; 2 Department of Health Promotion and Development, University of Pittsburgh School of Nursing, Pittsburgh, Pennsylvania, United States of America; 3 Department of Human Genetics, School of Public Health, University of Pittsburgh, Pittsburgh, Pennsylvania, United States of America; 4 Department of Biostatistics, School of Public Health, University of Pittsburgh, Pittsburgh, Pennsylvania, United States of America; 5 Center for Surgery and Public Health, Brigham and Women’s Hospital, Boston, Massachusetts, United States of America; 6 Family Health Clinic, Apia, Samoa; 7 Naseri & Associates Health Consultancy Firm, Apia, Samoa; 8 Lutia I Puava Ae Mapu I Fagalele, Apia, Samoa; 9 Department of Global and Community Health, College of Public Health, George Mason University, Fairfax, Virginia, United States of America; 10 Division of Endocrinology and Metabolism, Division of Medicine, University of Pittsburgh, Pittsburgh, Pennsylvania, United States of America; 11 School of Medicine, Oceania University of Medicine, Apia, Samoa; 12 Department of Epidemiology, School of Public Health, International Health Institute, Brown University, Providence, Rhode Island, United States of America; 13 Department of Anthropology, Brown University, Providence, Rhode Island, United States of America; UCL: University College London, UNITED KINGDOM OF GREAT BRITAIN AND NORTHERN IRELAND

## Abstract

Understanding psychosocial determinants of health is critical in understudied, high-risk populations, where such knowledge could have a major impact on health outcomes. In Samoa, where diabetes prevalence has rapidly increased over 30 years, associations between psychosocial factors and diabetes remain poorly understood. The purpose of our study was to identify psychosocial factors associated with HbA1c among Samoan adults while accounting for established correlates of HbA1c including age, body composition, and genetic and behavioral risk factors. Participants (n = 349 adult Samoans; 53% female) who were not receiving diabetes care were selectively sampled from the *Soifua Manuia* (2017 – 2019) study. Multiple linear regression with backwards elimination and a bootstrapping stability investigation was used to assess associations between HbA1c and participant demographics, genetics (rs373863828 A allele in *CREBRF*), biological, behavioral, and psychosocial variables (i.e., self-esteem, self-efficacy, social support, health related quality of life, perceived social conflict, food security). The rs373863828 genotype and age were statistically significantly associated with HbA1c—more copies of the rs373863828 A allele and younger age were associated with lower HbA1c. While not statistically significant based on confidence intervals, those with greater self-efficacy tended to have lower HbA1c, while those with higher social support tended to have higher HbA1c. Based on the frequent appearances of psychosocial factors in our models, we advocate for increased awareness and promotion of psychosocial health, and health promoting social support in Samoa. Integrating psychosocial and mental health awareness with standard biomedical protocols and health promotion materials provides an important and empowering means of diabetes prevention and management.

## Introduction

Psychosocial health is instrumental in chronic disease prevention and management, particularly in Type 2 diabetes (henceforth referred to as diabetes). Among adults with diagnosed diabetes, exposure to adverse psychosocial factors is associated with poorer glycemic control [1] and poorer quality of life [2]. Additionally, individuals with prediabetes, undiagnosed diabetes, and diagnosed diabetes are more likely to suffer from anxiety and depression compared to the general population [3,4]. In contrast, enhanced psychosocial health has been associated with better glycemic control and self-efficacy [5], and lower risk of comorbidities and chronic disease risk among people living with diabetes [6]. For those without a diabetes diagnosis, enhanced psychosocial health is associated with a lower risk of developing prediabetes [7] or progressing to diabetes [6]. Psychosocial factors are essential components of improving well-being and disease-related outcomes among those living with or at risk for diabetes [8,9]; however, these factors remain understudied as modifiable risk factors of cardiometabolic health [10–12] and are in turn under-integrated within management and prevention strategies [13].

There is a pressing need for increased assessments of how psychosocial factors are associated with diabetes risk in low and middle-income countries (LMICs); these locations currently comprise 80% of those living with diabetes worldwide [14]. While an obesity risk variant occurring at a higher frequency among Polynesian and Micronesian populations has been recently identified and is paradoxically associated with lower odds of type 2 diabetes (The A allele of rs373863828 in CREB3 regulatory factor) [15], diabetes prevalence in Samoa has continued to increase over the last 30 years largely due to rapid shifts in the economy, lifestyle, and an ongoing nutrition transition [16–19].

Psychosocial health in general, along with its associations to diabetes risk remains understudied in the Pacific and could provide important insight into the continuing rise in diabetes incidence among these groups. Research into psychosocial health conducted in the 1990s among Samoan adults in Samoa, American Samoa, and Hawai’i, identified that stress was relatively lower among the participants in Samoa, compared to those from American Samoa and Hawai’i. The increased stress among the latter groups was largely attributed to larger (non-kin) social networks, and increased adoption of ‘Western’ lifestyles (i.e., increased alcohol consumption, less engagement in traditional employment, and increased access to market-based economies) as compared to Samoa at that time [20]. Recent work has also examined psychosocial health among diabetes intervention participants in American Samoa, and found that perceived diabetes control, perceived benefits of diabetes control, and patient activation measures were not statistically associated with HbA1c [21]. As Samoa’s economic, social, food, and cardiometabolic risk environments continue to rapidly change [22–24], there is a vital need to understand how psychosocial factors among adults may be associated with cardiometabolic risk, and how psychosocial health may be leveraged to optimally benefit health in this setting.

The purpose of our study is to use a data-driven analytical approach to identify psychosocial factors associated with HbA1c among Samoan adults not currently receiving diabetes care while also considering established correlates such as age, population-specific genetic risk factors, body composition, and behavioral risk factors. We aim to explore whether the factors that were most strongly associated with HbA1c varied between sexes. This analysis seeks to pave the way for the development of more comprehensive, patient-empowered, and integrated strategies for diabetes prevention and management, and ultimately improve patient outcomes.

## Method

### Setting

The Independent State of Samoa is a Polynesian country located in the South Pacific Ocean. Much of the population of 207,501 [25] resides on the islands of ‘Upolu and Savai’i, with >70% residing on ‘Upolu, where data collection occurred. This island is made up of three census regions, the Apia Urban Area (AUA; urban), Northwest ‘Upolu (NWU; peri-urban); and the Rest of ‘Upolu (ROU; rural). Between 1978 and 2013, national age-standardized diabetes prevalence increased from 1.2% to 19.6% among men, and 2.2% to 19.5% among women [26]. The most recent Global Nutrition Report-- using data from the NCD Risk Factor Collaboration-- projected that by 2019, an estimated 31.0% of adult women and 26.9% of adult men would have diabetes [27]; however, updated nationally representative surveys and analyses are still needed to determine the accuracy of prior model predictions.

### Participant characteristics and data collection

Participants were from the *Soifua Manuia* (Good Health) study, conducted between August 2017- February 2019 [28]. The study was a follow up of participants who had previously taken part in a 2010 genome-wide association study (GWAS) [15]. The *Soifua Manuia* study (n = 519 participants; 32-72 years from the island of ‘Upolu) aimed to explore the impact of a missense variant (*CREBRF* rs373863828*)* which was identified in the 2010 GWAS and has a positive association with BMI, but a negative association with fasting glucose among Samoan adults [15]. Participants included in this analysis were those who did not report a diabetes diagnosis and subsequently were not taking any diabetes-related medications and presumably were not engaged in physician-prescribed diabetes self-management activities. All participants self-identified as Samoan (confirmed by genetic analyses [15]) and, given the focus on the rs373863828 variant, the follow up study included intentional oversampling of those with the *CREBRF* obesity-risk allele (A). Detailed inclusion and exclusion criteria can be found elsewhere [28,29]. Participants completed anthropometric measures, questionnaires, and biochemical assessments.

#### Ethics statement.

Consent documents were provided to participants in both Samoan and English, and the study objectives and protocol were explained to participants by Samoan Research Assistants. Following an assessment of their understanding of study expectations, participants provided written informed consent. Ethical approvals for this study were obtained from Yale and Brown Universities, the University of Pittsburgh, and the Health Research Committee of the Samoan Ministry of Health. Yale served as the IRB of record, IRB#1604017547.

#### CREBRF.

*CREBRF* rs373863828 genotype data were collected using a TaqMan assay (previously described [15]) and modeled using additive coding based on number of minor allele (A) copies.

#### Anthropometric measures.

Weight and height were measured in duplicate to the nearest 0.1 kg and 0.1 cm, respectively (Tanita HD 351, Tanita Corporation of America; SECA 213, Seca GmbH & Co), averaged, and used to compute BMI. Abdominal circumference was measured in duplicate to the nearest 0.1 cm and averaged for use in analyses. Fat mass was calculated using a highly accurate (r^2^=0.95 with DXA-derived fat mass) Polynesian-specific anthropometric equation based on age, sex, height, and weight [30]; fat mass index (kg/m^2^) was calculated by dividing fat mass by height squared. Capillary blood HbA1c was measured using a finger-stick point-of-care testing device (DCA vantage analyzer, Siemens Healthcare GmbH). Participants with HbA1c values ≥ 6.5% were classified as having diabetes, 5.7% to < 6.5% as having prediabetes, and < 5.7% as having glycated hemoglobin values indicative of not having diabetes [31].

#### Questionnaires: Demographic characteristics, behaviors, and socioeconomic factors.

Age, sex, and census region of residence were self-reported. A household asset score, a proxy for socioeconomic status used previously in Samoa, was comprised of an additive score of items owned from an 18-item household inventory (refrigerator, freezer, portable stereo/MP3 player, etc.) [28]. Other self-reported sociodemographic factors included years of education and relationship status (partnered/not partnered). The Global Physical Activity Questionnaire was used to estimate self-reported daily moderate-to-vigorous physical activity (MVPA) minutes [32]. Additional behavioral factors included smoking (current use of cigarettes, tobacco, or pipe; yes/no), consumption of alcohol in the last 12 months (yes/no) [33,34] and adherence to dietary patterns (classified as mixed traditional, modern, and health-conscious). Dietary intake was assessed using a 104-item food frequency questionnaire validated for use among adults in the Samoan setting [35]. Factor analysis with the principal component method was used to derive dietary patterns in a process like that described by Wang et al.[36]. Briefly, individual food items were grouped based on nutrient profiles and culinary use in Samoa and the sum of individual food frequencies were adjusted for total energy intake with the residual method [37]. Factors were rotated with orthogonal transformation to ensure a lack of correlation and the number of factors retained (three) was determined based on visual examination of the scree plot, eigenvalues, and the proportion of variance explained. Factor scores for each dietary pattern were used as continuous variables in analyses, with higher scores indicating greater dietary pattern adherence. The mixed traditional pattern included higher intakes of locally cultivated items such as coconut products and seafood, and lower intakes of processed items such as breads, cakes, and traditional staples including yams, taro, breadfruit, and bananas. The modern pattern was typified by higher intakes of processed items including snacks, confections and desserts, nuts, poultry, and jams. The health-conscious diet was characterized by higher intake of vegetables, fruits, and soups, and lower intakes of protein (including red meat and poultry).

#### Questionnaires: Psychosocial factors.

Perceived stress was measured using the 10-item Cohen’s Perceived Stress Scale (scores from 0 to 40) [38] and social conflict was measured using the Perceived Social Conflict Score (scores from 6 to 30) [39]; higher scores indicated higher perceived stress and social conflict, respectively. Self-esteem was measured using the Rosenberg Self Esteem Questionnaire, a 10-item Likert scale measure that assesses global self-worth and both positive and negative feelings about oneself, with higher scores indicating more positive self-esteem (scores from 10 to 40) [40]. Discrimination was assessed using the total additive score derived from the Everyday Discrimination questionnaire (scores from 0 to 50) [41], a 21-item measure that subjectively assesses experiences of discrimination; participants reported the frequency with which they experienced routine mistreatment (e.g., being treated with less respect than others or being threatened), where higher scores indicated more frequent experiences of everyday discrimination. Social support was measured using the total score of the Multidimensional Scale of Perceived Social Support (MSPSS, scores 1 to 5), which includes questions related to support from family, friends, and significant others, with higher scores indicating more perceived social support [42]. Food security was measured with the Household Food Insecurity Access Scale (scores 0 to 27) [43]. General self-efficacy was measured using the General Self Efficacy subscale of the Self Efficacy Scale [44,45]. The subscale consists of a 17-item scale with higher scores indicating greater perceived self-efficacy (scores from 17 – 85 when using a 5-point Likert scale). The Short Form-8 (SF-8) was used to assess perceived physical- and mental health-related quality of life using the Physical Health Component Score and Mental Health Component Score summary measures; both result in values of 0–100 with higher scores indicating better perceived health-related quality of life [46]. All questionnaires were translated in Samoan and back translated into English by trained bilingual members of the research team. Questionnaires were pilot tested for understanding and face validity before use.

### Statistical analyses

All statistical analyses were performed in R version 4.3.2. We used standard descriptive statistics to summarize our data, including means (standard deviation, SD), medians (interquartile range, IQR), and ranges for continuous variables, as well as frequency counts and percentages for categorical variables. We assessed patterns of missing data and, where appropriate, missing data for continuous summary score variables with a missingness rate ranging from 5% to 10% (household asset score, SF-8 Physical and Mental Health, Cohen’s Perceived Stress Scale, Perceived Conflict Score, and MSPSS) were imputed using within-domain imputation [47] as detailed in our prior work [48]. Sensitivity analyses were performed to evaluate the impact of imputation on our results.

To comprehensively analyze associations between HbA1c (dependent variable) and participant characteristics (independent variables), we applied a rigorous data-driven approach via multiple linear regression with backward elimination based on the Akaike information criterion (AIC) and a bootstrapping stability investigation [49]. This approach included evaluation of (1) a global model which included all *a priori* selected candidate variables (described above) and (2) a “selected” model which included variables chosen through backward elimination. Recognizing the potential for bias associated with use of the same dataset for both variable selection and post-selection inference, we conducted a bootstrap stability analysis [49]. Specifically, we repeated the backward elimination selection procedure across 1000 bootstrapped resamples of the data. Based on these results we added (3) a final “inference” model which reported the median of the test statistics across 1000 resamples. The bootstrapping approach applied here is more robust than traditional linear regression and includes built-in adjustment for multiple comparisons. Across all models, we reported the regression coefficients ($\hat{\beta }$) and their corresponding 95% confidence intervals (CI). We also reported the standardized regression coefficients (.$Std.\hat{\beta }$.) for the bootstrapped models to facilitate comparison of variable effect sizes. We were also able to calculate several measures of variable importance and selection stability such as variable-specific bootstrap-inclusion frequencies as well as measures of variation in regression coefficients around the full model estimates. We assessed variables having greater than 60% variable inclusion frequency in the bootstrap models as meaningful to our findings. Residual analysis, collinearity checks, and influence diagnostics, as well as sensitivity analyses were used to assess the selected model’s performance. To fully document the details of our analyses, we provide our analytical code (S1 Code).

We performed our analyses on the entire participant cohort and examined the data stratified by sex. Of note, we included (i.e., forced) age, sex, BMI, and rs373863828 genotype variables as covariates across all bootstrapped resamples based on *a priori* knowledge of the Samoan population or to account for study design considerations.

## Results

### Sample description

To be included in the applied data-driven multivariable modeling approach, individuals were required to have complete data for all 24 variables of interest post-imputation, resulting in a sample size of n=349 (53% female). The sample had a mean ( ±  SD) age of 51.3 ( ±  9.8) years and BMI of 36.1 ( ±  7.9) kg/m^2^ (Table 1). The median HbA1c was 6.0% (±0.7); approximately 23% and 59% of the sample could be categorized as having HbA1c values consistent with diabetes and prediabetes, respectively. Except for diabetes medication use (an exclusion criterion), no statistically significant differences were observed between the study sample and the larger sample of n=519 (S1 Table).

**Table 1:** Sample characteristics.

Characteristics	Value	Total (n = 349)	Male (n = 165)	Female (n = 184)
*Demographics*				
Age (years)	Mean (SD)	51.3 (9.8)	52.7 (10.1)	50.1 (9.3)
	Range	30.7–72.5	30.7–72.5	32.7–71.9
Sex (%)	Male (%)	165 (47%)		
	Female (%)	184 (53%)		
Census Region n (%)	Apia Urban Area (AUA) (%)	72 (21.0%)	33 (20.0%)	39 (21.0%)
	Northwest ‘Upolu (NWU) (%)	137 (39%)	68 (41%)	69 (38%)
	Rest of ‘Upolu (ROU) (%)	140 (40%)	64 (39%)	76 (41%)
Education (Years)	Mean (SD)	11.3 (2.7)	10.8 (3.2)	11.6 (2.1)
	Range	0–19	0–19	4–17
Number of Household assets (socioeconomic status indicator)	Mean (SD)	7.9 (3.9)	8.1 (4.3)	7.7 (3.6)
	Range	0–18	1–17	0–18
Relationship n (%)	Not partnered n (%)	60 (17.0%)	30 (18.0%)	30 (16.0%)
	Partnered n (%)	289 (83.0%)	135 (82.0%)	154 (84.0%)
*Diabetes Risk*				
HbA1c (%)	Median (IQR)	6.0 (0.7)	5.9 (0.4)	6.0 (0.8)
	Range	5.1–13.5	5.3–13.3	5.1–13.5
Categorical HbA1c n (%)	< 5.7% (not indicative of prediabetes or diabetes)	60 (17.0%)	32 (19.0%)	28 (15.0%)
	5.7–<6.5% (indicative of prediabetes)	207 (59.0%)	98 (59.0%)	109 (59.0%)
	≥ 6.5% (indicative of diabetes)	82 (23.0%)	35 (21.0%)	47 (26.0%)
CREBRF genotype n (%)	GG	151 (43.0%)	72 (44.0%)	79 (43.0%)
	AG	139 (40.0%)	65 (39.0%)	74 (40.0%)
	AA	59 (17.0%)	28 (17.0%)	31 (17.0%)
*Body Composition*				
Body Mass Index (BMI; kg/m^2^)	Mean (SD)	36.1 (7.9)	33.3 (6.7)	38.6 (7.9)
	Range	20.2–76.7	20.2–60.1	20.6–76.7
Abdominal Circumference (cm)	Mean (SD)	113.7 (16.4)	109.3 (16.5)	117.6 (15.3)
	Range	76.5–178.6	76.5–167.5	79.9–178.6
Fat mass index (kg/m^2^)	Mean (SD)	4.0 (5.9)	10.3 (4.4)	17.3 (5.1)
	Range	1.9–42.3	1.9–27.5	6.5–42.3
*Psychosocial Factors*				
SF-8 Physical Health	Mean (SD)	43.8 (8.3)	44.1 (8.4)	43.6 (8.3)
	Range	20.0–63.1	20.0–63.1	22.9–62.4
SF-8 Mental Health	Mean (SD)	46.7 (9.6)	47.0 (9.8)	46.4 (9.4)
	Range	15.2–66.3	24.5–65.4	15.2–66.3
Self-efficacy	Mean (SD)	52.8 (5.6)	53.8 (6.1)	51.9 (5.0)
	Range	40–76.0	41.0–76.0	40.0–65.0
Social Support	Mean (SD)	3.9 (0.5)	4.0 (0.5)	3.9 (0.5)
	Range	1.7–5.0	2.2–5.0	1.7–5.0
Self-esteem	Mean (SD)	30.7 (3.9)	30.8 (4.3)	30.6 (3.6)
	Range	10–40	10–40	10–40
Stress	Mean (SD)	17.8 (5.6)	17.1 (6.1)	18.4 (5.0)
	Range	0–32.0	0–32.0	0–28.0
Perceived Discrimination	Median (IQR)	2.0 (5.0)	2.0 (5.0)	2.0 (5.0)
	Range	0–24.0	0–24.0	0–24.0
Food security	Mean (SD)	0.35 (1.25)	0.24 (0.95)	0.44 (1.47)
	Range	0–9.0	0–8.0	0–9.0
*Behavioral Factors*				
Alcohol Use n (%)	No	318 (91.0%)	139 (84.0%)	179 (97.0%)
	Yes	31 (8.9)	26 (16.0%)	5 (2.7%)
Current smoker n (%)	No	208 (60%)	76 (46%)	132 (72%)
	Yes	141 (40%)	89 (54%)	52 (28%)
Physical Activity n (%)	0 MVPA minutes/week	247 (71%)	97 (59.0%)	150 (2.0%)
	> 0 MVPA minutes/week	102 (29%)	68 (41.0%)	34 (18.0%)

Dietary pattern data are not included in this table due to their nature as factor scores. These scores are centered on 0 with a standard deviation of 1.

### Overall model results

In the bootstrapped model for the total sample; age [median of the bootstrapped estimates, $\hat{\beta_{b}}$ = 0.020 (95% CI 0.005 to 0.040) Std. $\hat{\beta_{b}}=0.152$] was positively associated with HbA1c; more copies of the rs373863828 A allele [$\hat{\beta_{b}}$ = -0.339 (95% CI -0.514 to -0.157) Std. $\hat{\beta_{b}}=-0.187$] was negatively associated (Table 2).

**Table 2:** Results of association testing including global model, selected model, and final model with bootstrap-derived quantities for multi-model inference and assessment of model uncertainty (n=349).

	Global Model				Selected Model					Final Bootstrap Estimates			
	$\hat{\beta_{g}}$	2.5th	97.5th	Bootstrap inclusion frequency	$\hat{\beta_{s}}$	2.5th	97.5th	RMSD Ratio	RC Bias	$\hat{\beta_{b}}$	2.5th	97.5th	Std. $\hat{\beta_{b}}$
(Intercept)	0.243	–7.994	8.480	100	1.469	–7.994	8.480	0.753	246.997	1.085	–7.156	6.768	
**Age**	**0.022**	**0.000**	**0.043**	**100**	**0.018**	**0.000**	**0.043**	**0.848**	–**1.889**	**0.020**	**0.005**	**0.040**	**0.152**
Sex, Female (Ref=Male)	–0.154	–2.430	2.123	100	–0.285	–2.430	2.123	0.616	26.538	–0.261	–1.968	1.895	–0.101
BMI	0.001	–0.411	0.414	100	–0.025	–0.411	0.414	0.594	–681.585	–0.023	–0.348	0.345	–0.135
***CREBRF* rs373863828**	**–0.336**	**–0.528**	**–0.145**	**100**	**–0.354**	**–0.528**	**–0.145**	**0.904**	**0.810**	**–0.339**	**–0.514**	**–0.157**	**–0.187**
Abdominal circumference	0.034	0.012	0.056	91.375	0.035	0.012	0.056	1.373	6.903	0.035	0	0.061	0.426
Social support (MSPSS)	0.315	–0.001	0.630	77.375	0.326	–0.001	0.630	1.157	17.537	0.320	0	0.609	0.111
Stress (PSS)	0.033	0.002	0.065	76.875	0.030	0.002	0.065	1.269	14.704	0.032	0	0.066	0.132
Self–efficacy	–0.024	–0.052	0.004	68.5	–0.020	–0.052	0.004	1.112	22.469	–0.024	–0.046	0	–0.102
Dietary pattern, Mixed traditional	–0.121	–0.281	0.039	57.25	–0.113	–0.281	0.039	1.191	42.313	–0.120	–0.275	0	–0.086
SF-8 Mental health	0.013	–0.004	0.031	56.75	0.012	–0.004	0.031	1.359	43.282	0.013	0	0.034	0.095
Census region, NWU (Ref=AUA)	0.144	–0.233	0.521	47.125				0.921	36.258	0	–0.147	0.485	0
Census region, ROU (Ref=AUA)	0.284	–0.098	0.666	47.125				1.283	38.985	0	0	0.635	0
Years of education	0.024	–0.034	0.083	34.625				1.035	118.630	0	0	0.082	0
Alcohol, Yes (Ref=No)	0.192	–0.313	0.698	33				1.109	127.802	0	–0.377	0.806	0
SF–8 Physical health	0.006	–0.012	0.025	29.375				0.996	131.483	0	–0.012	0.025	0
Perceived discrimination	–0.015	–0.047	0.018	27.75				0.944	101.291	0	–0.041	0	0
Socioeconomic resources	–0.009	–0.048	0.030	23.875				0.914	200.084	0	–0.051	0.029	0
Smoking, Yes (Ref=No)	0.055	–0.234	0.344	22.375				0.898	148.516	0	–0.255	0.363	0
Dietary pattern, Health conscious	0.034	–0.117	0.184	22				0.803	195.280	0	–0.103	0.172	0
Perceived social conflict	–0.001	–0.026	0.025	21.875				0.872	984.959	0	–0.029	0.027	0
Food security	0.035	–0.080	0.150	20.25				0.853	175.268	0	0	0.141	0
Partnered, Yes (Ref=No)	–0.013	–0.384	0.358	19.375				0.835	299.967	0	–0.435	0.340	0
Self-esteem	0.006	–0.032	0.043	16.75				0.709	273.698	0	–0.025	0.038	0
MVPA minutes/week, >0 (Ref=0)	–0.017	–0.344	0.309	14				0.673	364.173	0	–0.323	0.268	0
Dietary pattern, Modern	0.042	–0.118	0.202	12.625				0.708	196.525	0	0	0.173	0
Fat mass index	–0.039	–0.666	0.587	11.625				0.595	387.423	0	–0.577	0.473	0

Global and selected models presented alongside the final bootstrap model to assess the stability of the estimates and variable selection bias; global model shows the estimates and confidence intervals when all variables of interest are included in the model; selected model, selected via backward elimination with a significance level of 0.157 (AIC selection); final bootstrap estimates, indicate the bootstrap median estimate across all bootstrapped iterations; $\hat{\beta }$, unstandardized coefficient estimates with subscripts of g, s, and b corresponding to the global, selected, and bootstrapped models, respectively; 2.5th and 97.5th percentiles interpreted as limits of 95% confidence intervals; * symbol indicates variables included in all models based on *a priori* knowledge of the Samoan population or to adjust for study design strategy; estimated shrinkage factor of model 0.693; selected model frequency 0.1%; all variance inflation factors of selected model <1.5, with the exception of BMI and abdominal circumference, with variance inflation factors of 7.4 and 6.9, respectively; RMSD, root mean squared difference; global model, R^2^=18.7, adjusted R^2^=12.4; selected model, R^2^=16.6, adjusted R^2^=14.1; bolded values indicate statistical significance based on bootstrap confidence intervals.

Several other variables were frequently selected (> 60% of the iterations) via backwards elimination, though they were not ultimately found to be significantly associated with HbA1c in the final bootstrapped results based on confidence intervals. Variables that were positively associated with HbA1c included social support which showed the greatest (positive) relationship with HbA1c [$\hat{\beta_{b}}$ = 0.320 (95% CI 0.000 to 0.609); Std. $\hat{\beta_{b}}=0.111$], abdominal circumference [$\hat{\beta_{b}}$ = 0.035 (95% CI 0.000 to 0.061); Std. $\hat{\beta_{b}}=0.426$], stress [$\hat{\beta_{b}}$ = 0.032 (95% CI 0.000 to 0.066); Std. $\hat{\beta_{b}}=0.132$], and mental health-related quality of life (SF-8) [$\hat{\beta_{b}}$ = 0.013 (95% CI -0.000 to 0.034); Std. $\hat{\beta_{b}}=0.095$]. Frequently included variables that were negatively associated with HbA1c included traditional dietary pattern factor scores [($\hat{\beta_{b}}$ = -0.120 (95% CI -0.275 to 0.000); Std. $\hat{\beta_{b}}=0.086$], and self-efficacy [$\hat{\beta_{b}}$ = 0-0.024 (95% CI -0.046 to 0.000); Std. $\hat{\beta_{b}}=-0.102$]. In sensitivity analyses evaluating the impact of imputation, no differences were observed in the variables identified as most critical, nor in the directions or general magnitudes of the effects.

#### Sex-stratified model results: Males.

Among male participants, more copies of the rs373863828 A allele was associated with lower HbA1c [$\hat{\beta_{b}}$ = -0.419 (-0.702 to -0.134); Std. $\hat{\beta_{b}}=-0.204$], and greater abdominal circumference was associated with higher HbA1c [$\hat{\beta_{b}}$ = 0.084 (0.032 to 0.132); Std. $\hat{\beta_{b}}=0.917$]. While other associations were not statistically significant based on confidence intervals, there were several variables frequently selected (> 60%) by backwards elimination, all of which were psychosocial factors. Of the psychosocial variables, mental health-related quality of life (SF-8) [$\hat{\beta_{b}}$ = 0.025 (0.000 to 0.057); Std. $\hat{\beta_{b}}=0.168$], social support [$\hat{\beta_{b}}$ = 0.574 (0.000 to 1.184); Std. $\hat{\beta_{b}}=0.175$], stress [$\hat{\beta_{b}}$ = 0.0.056 (0.000 to 0.116); Std. $\hat{\beta_{b}}=0.226$], and food security [$\hat{\beta_{b}}$ = 0.223 (0.000 to 0.469); Std. $\hat{\beta_{b}}=0.140$], had similar effect sizes and all were positively associated with HbA1c. (S2 Table)

#### Sex-stratified model results: Females.

Among female participants, age was positively associated with HbA1c [$\hat{\beta_{b}}$ = 0.042 (95% CI 0.017 to 0.086); Std. $\hat{\beta_{b}}=0.351$] whereas more copies of the rs373863828 A allele was negatively associated with HbA1c [$\hat{\beta_{b}}$ = ‒0.244 (95% CI -0.437 to -0.067); Std. $\hat{\beta_{b}}=-0.164$]. Notably, except for self-efficacy [$\hat{\beta_{b}}$ = ‒0.021 (95% CI: -0.060 to 0.000); Std. $\hat{\beta_{b}}=-0.107$], psychosocial factors (none of which were statistically significant based on confidence intervals) appeared to be less important to, and had lower effect sizes on, HbA1c for female participants than for male participants, as evidenced by generally lower bootstrap inclusion frequencies. Instead, frequently selected variables (>60%) via backward elimination included mixed traditional dietary pattern factor scores (negatively associated with HbA1c; [$\hat{\beta_{b}}$ = ‒0.178 (-0.423 to 0.000); Std. $\hat{\beta_{b}}=-0.142$]), along with education [$\hat{\beta_{b}}$ = 0.088 (0.000 to 0.218); Std. $\hat{\beta_{b}}=0.167$], and self-reported smoking (both positively associated with HbA1c; [$\hat{\beta_{b}}$ = 0.290 (0.000 to 0.751); Std. $\hat{\beta_{b}}$ = 0.124) (S3 Table).

## Discussion

Our goal was to identify and characterize associations between psychosocial variables and HbA1c among Samoan adults. Our results reveal several important correlates of HbA1c including genetic (number of copies of the rs373863828 A allele*)*, biological (age and abdominal circumference), behavioral (mixed traditional dietary adherence), and psychosocial factors (i.e., social support, self-efficacy, and for some, mental health-related quality of life).

The rs373863828 A allele had the strongest association with HbA1c compared to all other measured variables, re-affirming findings from prior studies in this setting [15,50], and others [50–52], of its importance for glucose management among Pacific Islanders, even when controlling for other risk factors. Ongoing investigations into the variant’s mechanism of action will be important for informing future intervention approaches.

While their effects on HbA1c were smaller than copies of the rs373863828 A allele, established biological risk factors including older age and greater abdominal circumference [53,54], were associated with elevated glycemia and poor cardiometabolic health. Participants with greater adherence to a mixed traditional diet that comprised a substantially greater intake of locally cultivated items such as coconut products and seafood tended to have lower HbA1c as expected compared to greater adherence to ‘modern diets’ that included a greater intake of processed items [55,56]. The health-conscious diet was not frequently selected across bootstrapped resample (possibly due to the diet comprising greater amounts of carbohydrates, and lower amounts of protein than the mixed-traditional diet), but further study is needed.

While not demonstrating statistical significance, several psychosocial factors were frequently selected in our models and indicated comparatively minor yet potentially meaningful differences in HbA1c.

### Social support

Our models found that participants with greater perceived social support tended to have higher HbA1c. Social support is most often associated with *better* cardiometabolic health including glucose management among those living with diabetes [57–59]. Increased social support is postulated to help affected individuals by offering emotional, financial, material, or cognitive guidance [60], and may provide avenues that help patients reduce stress, engage in routines, and cope with their diagnoses [61]. However, prior work in this region has demonstrated variable associations between social support and health. For example, among acculturated Samoan women living in Hawai’i and American Samoa, social support was found to be associated with lower blood pressure; however, among men in Samoa, American Samoa, and Hawai’i, larger social networks were associated with higher blood pressure [62]. Additionally, social integration has been found to be positively associated with lifestyle incongruity stress among Samoan adolescents [63].

There are several potential explanations for our results showing a positive trend between social support and higher HbA1c among individuals not yet diagnosed with diabetes. Firstly, family and community support are heavily engrained and valued within Samoan culture [64], especially for those who are considered vulnerable (elderly, very young, infirm – or perhaps, in this case, at risk of poor health). Factors often associated with diabetes risk such as other chronic conditions, and/or higher weight status [65], may have increased the likelihood of receiving social support. Secondly, Samoan culture has been characterized as ‘collectivist’ [66], and social support (perceived to be higher in collectivist societies [66]) in Samoa is frequently expressed and manifested through community and familial events that revolve around food [67–69]; it is possible individuals may feel hesitant to opt out of such activities. It is also possible that the social support experienced by participants may be unintentionally ‘obstructive’ [70] if the surrounding social network or individual themselves have reduced health or nutrition literacy [70], and does not fully understand the nutritional, physical, or emotional needs of the at-risk individual [71]. Notably, social support may be perceived to be externally constructed to a greater extent than self-esteem and self-efficacy. Our findings indicate a potential increased need and awareness for greater health literacy within the surrounding social networks of the participants.

While the small sample sizes of the stratified results preclude a comprehensive assessment of differences between the males and females, it is interesting to note that as a whole, a greater number of psychosocial health variables showed importance in the men; social support specifically was also positively associated (though not statistically significant) with higher Hba1c in the men, similar to the positive associations between larger social networks and blood pressure found previously in men of Samoan descent [62]. Ultimately, the relationship between social support and HbA1c should be examined using mixed methods to optimize and adapt supportive behaviors beneficial to cardiometabolic health, as it is a potentially modifiable factor that could have broad reach and impact among those at risk.

### Self-efficacy and health related quality of life

Those with greater self-efficacy tend to have better (lower) HbA1c values, as expected based on prior studies. Self-efficacy, notably, was the only psychosocial variable that showed importance and was frequently selected within the female model. Most research to date has focused on assessing self-efficacy in relation to diabetes self-management (including glycemia levels) among patients engaged in care, and these works have found similar associations [72,73]. In other studies of non-diagnosed individuals, higher self-efficacy has been found to be associated with greater knowledge of diabetes risk factors [74], and higher diabetes risk has been found to be associated with lower self-efficacy [75], patterns that could potentially be mirrored here with indirect effects on HbA1c. Further work is needed to understand how to best optimize heightened self-efficacy as a tool for diabetes prevention among high-risk groups.

Those with perceived better mental health-related quality of life tended to have worse (higher) HbA1c. This result is not unexpected in this sample given that some participants’ HbA1c did not meet the clinical thresholds for prediabetes or diabetes where one may expect an increased risk of experiencing diabetes-related symptoms, lifestyle, or financial burdens impacting daily life [76]. Even then, prior work among this sample has found that the presence of symptoms associated with hyperglycemia was not associated with adverse perceptions of health-related quality of life [77]. Furthermore, by design, participants were unaware of their glycemic status; therefore, those with HbA1c indicative of diabetes would be unlikely to be impacted by the emotional distress, stress, lifestyle changes, or uptake of an illness identity that can accompany a chronic disease diagnosis [78,79]. Prior work among Samoans has also identified that perceived health is ‘communal’ [69] and cannot be defined by binary categories of mental and physical health alone, but instead often comprises perceptions of social, spiritual, physical, and moral wellbeing [69]. Better perceptions of these other aspects of health-related quality of life may have superseded any perceptions of physical or mental distress. Additionally, the collectivist nature of Samoan culture, as noted previously [66], may provide a buffer against individual feelings of distress, resulting in improved perceptions of quality of life. Similar to findings reported elsewhere [80], our results indicate that culture may be an important factor shaping perceptions of psychosocial health among the sample.

Multiple factors are associated with cardiometabolic health in this setting; however, the frequent inclusion of psychosocial factors in our models indicates that efforts to bring awareness and empower those at risk to improve their psychosocial health should be promoted and integrated within biomedical care [81]. Our assessments, specifically among individuals not currently engaged in care or aware of their glycemic status, fills an important research gap, especially since it is estimated that approximately half of those living with diabetes worldwide remain undiagnosed [82–84]. Notably, given the observed HbA1c values in this sample, our findings indicate that there is likely a high prevalence of undiagnosed diabetes occurring among the participants. While improving access to screening and care is fundamental towards helping individuals manage their diabetes risk, ensuring patients and at-risk individuals have enhanced psychosocial wellbeing may also help strengthen the capacity for prevention and reduce future diabetes-related burdens, and complications in this setting and beyond.

### Strengths and limitations

Using a rigorous statistical approach, we have presented a holistic assessment of the multitude of correlates influencing HbA1c in this sample of adults in Samoa. The study is limited in several ways. Due to the sample size, and sampling strategy, we are unable to provide results that are representative of the correlates of HbA1c among the broader adult Samoan populace. Additionally, only the face validity of the questionnaires was tested in the cohort. Given our limited sample size, we were also unable to provide comprehensive analyses of differences that may be occurring between the sexes. We advocate for the expansion of psychosocial health indicators assessed in this setting. In addition, given the age range of participants within our study, and the rapid pace of changes impacting the lived and food environments in Samoa over the last 50 years, further research is needed to understand how age, and life experiences have impacted cardiometabolic risk and perceptions of physical and mental health within this setting. Greater inquiry into how psychosocial factors and stressors are culturally and socially defined, perceived, and embodied in Samoa is needed. Future research would also benefit from a greater understanding of how generational differences may be influencing perceptions of psychosocial health.

## Conclusion

A known gene variant had the strongest association with HbA1c among this sample of adult Samoans; however, due to the frequent appearance of psychosocial factors in our data-driven models, and their suggestive associations with cardiometabolic health, we advocate for increased awareness and promotion of psychosocial health and wellbeing in this setting. Strengthening psychosocial health, and health promoting social support and self-efficacy has the potential to provide individuals with greater agency and an empowering means of diabetes prevention and management.

## Supporting information

S1 Table Participant Characteristics of Parent Sample (2017–2019 Follow-up). (DOCX)

S2 Table Sex-stratified results, male. Results of association testing including global model, selected model, and final model with bootstrap-derived quantities for multi-model inference and assessment of model uncertainty, male subgroup (n=165).
(DOCX)

S3 Table Sex-stratified results, female. Results of association testing including global model, selected model, and final model with bootstrap-derived quantities for multi-model inference and assessment of model uncertainty, female subgroup (n=184).
(DOCX)

S1 Text Translated Abstract. Abstract in Samoan.
(DOCX)

S1 Code Analyses code. Executable analysis code and a simulated data set.
(DOCX)

S4 Table STROBE checklist. (DOCX)
