# Supplementary material for: Psychosocial correlates of HbA1c among adult Samoans without diabetes
Source: PLOS Ment Health. 2025 Feb 28;2(2):e0000196. doi: 10.1371/journal.pmen.0000196 (PMC12781966; doi:10.1371/journal.pmen.0000196)
Supplement: S1 Table — (DOCX) [file pmen.0000196.s001.docx]

| **S1 Table. Participant Characteristics of Parent Sample (2017 – 2019 Follow-up)** | | | |
| --- | --- | --- | --- |
| **Characteristic** | **All, N=519** | **Analysis subgroup, n = 349** | **p-value** |
| Age (years) |  |  |  |
| Mean (SD) | 52.2 (10.0) | 51.3 (9.8) | 0.190^1^ |
| Range | 30.7 to 72.7 | 30.7 to 72.5 |  |
| Sex, n (%) |  |  |  |
| Male | 233 (45%) | 165 (47%) | 0.478^2^ |
| Female | 286 (55%) | 184 (53%) |  |
| BMI (kg/m²) |  |  |  |
| Mean (SD) | 35.8 (7.7) | 36.1 (7.9) | 0.578^1^ |
| Range | 20.2 to 76.7 | 20.2 to 76.7 |  |
| Missing | 16 | 0 |  |
| Abdominal Circumference |  |  |  |
| Mean (SD) | 113.4 (15.9) | 113.7 (16.4) | 0.788^1^ |
| Range | 76.5 to 178.6 | 76.5 to 178.6 |  |
| Missing | 16 | 0 |  |
| Fat mass index |  |  |  |
| Mean (SD) | 13.8 (5.7) | 14.0 (5.9) | 0.617^1^ |
| Range | 1.87 to 42.3 | 1.9 to 42.3 |  |
| Missing | 16 | 0 |  |
| SF-8 Physical Health |  |  |  |
| Mean (SD) | 43.4 (8.4) | 43.8 (8.3) | 0.490^1^ |
| Range | 17.4 to 63.1 | 20.0 to 63.1 |  |
| Missing | 28 | 0 |  |
| HbA1C |  |  |  |
| Median (IQR) | 6 (0.9) | 6.0 (0.7) | 0.998^3^ |
| Range | 4.9 to 14.0 | 5.1 to 13.5 |  |
| Missing | 55 | 0 |  |
| Categorical HbA1c, n (%) |  |  |  |
| <5.7% | 76 (16%) | 60 (17%) | 0.136^2^ |
| 5.7% to <6.5% | 250 (54%) | 207 (59%) |  |
| $\geq$6.5% | 138 (30%) | 82 (23%) |  |
| Missing | 55 | 0 |  |
| Diabetes medication, n (%) |  |  |  |
| No | 469 (90.4) | 349 (100) | **0.0001^4^** |
| Yes | 50 (9.6) | 0 |  |
| *CREBRF* genotype, n (%) |  |  |  |
| GG | 224 (43%) | 151 (43%) | 0.237^2^ |
| AG | 201 (39%) | 139 (40%) |  |
| AA | 94 (18%) | 59 (17%) |  |
| Census Region, n (%) |  |  |  |
| AUA | 116 (22%) | 72 (21%) | 0.320^2^ |
| NWU | 221 (43%) | 137 (39%) |  |
| ROU | 182 (35%) | 140 (40%) |  |
| Education (Years) |  |  |  |
| Mean (SD) | 11.2 (2.7) | 11.3 (2.7) | 0.593^1^ |
| Range | 0 to 20 | 0 to 19 |  |
| Missing | 1 | 0 |  |
| Socioeconomic status (No. household assets) |  |  |  |
| Mean (SD) | 8.0 (4.0) | 7.9 (3.9) | 0.715^1^ |
| Range | 0 to 18 | 0 to 18 |  |
| Missing | 3 | 0 |  |
| Relationship, n (%) |  |  |  |
| Not partnered | 95 (18%) | 60 (17%) | 0.187^2^ |
| Partnered | 423 (82%) | 289 (83%) |  |
| Missing | 1 | 0 |  |
| Physical activity, n (%) |  |  |  |
| 0 minutes/week | 381 (74%) | 247 (71%) | 0.892^2^ |
| >0 minutes/week | 136 (26%) | 102 (29%) |  |
| Missing | 2 | 0 |  |
| Smoking, n (%) |  |  |  |
| No | 320 (62%) | 208 (60%) | 0.415^2^ |
| Yes | 198 (38%) | 141 (40%) |  |
| Missing | 1 | 0 |  |
| Alcohol use, n (%) |  |  |  |
| No | 478 (92%) | 318 (91%) | 0.269^2^ |
| Yes | 41 (7.9%) | 31 (8.9%) |  |
| Stress |  |  |  |
| Mean (SD) | 17.8 (5.5) | 17.8 (5.6) | 1.0^1^ |
| Range | 0 to 32 | 0 to 32 |  |
| Missing | 28 | 0 |  |
| Perceived discrimination |  |  |  |
| Median (IQR) | 2.0 (5.0) | 2.0 (5.0) | 1.0^1^ |
| Range | 0 to 35 | 0 to 24 |  |
| Missing | 37 | 0 |  |
| Self esteem |  |  |  |
| Mean (SD) | 30.7 (4.1) | 30.7 (3.9) | 0.991^1^ |
| Range | 10 to 40 | 10 to 40 |  |
| Missing | 47 | 0 |  |
| Social support |  |  |  |
| Mean (SD) | 3.9 (0.5) | 3.9 (0.5) | 1.0^1^ |
| Range | 1.6 to 5 | 1.7 to 5 |  |
| Missing | 28 | 0 |  |
| SF-8 Mental Health |  |  |  |
| Mean (SD) | 46.7 (9.8) | 46.7 (9.6) | 1.0^1^ |
| Range | 15.2 to 66.7 | 15.2 to 66.3 |  |
| Missing | 28 | 0 |  |
| Self-efficacy |  |  |  |
| Mean (SD) | 52.5 (5.6) | 52.8 (5.6) | 0.439^1^ |
| Range | 35 to 76 | 40 to 76 |  |
| Missing | 63 | 0 |  |
| Food security |  |  |  |
| Mean (SD) | 0.49 (1.56) | 0.35 (1.25) | 0.162^1^ |
| Range | 0 to 9 | 0 to 9 |  |
| Missing | 28 | 0 |  |

^1^T-test; ^2^Chi-square test; ^3^Mann-Whitney U Test; ^4^Fisher’s Exact Test.

Note: Dietary pattern data are not included in this table due to their nature as factor scores. These scores are centered on 0 with a standard deviation of 1, making them not directly interpretable in the context of mean (SD) reporting.
