# Supplementary material for: Psychosocial correlates of HbA1c among adult Samoans without diabetes
Source: PLOS Ment Health. 2025 Feb 28;2(2):e0000196. doi: 10.1371/journal.pmen.0000196 (PMC12781966; doi:10.1371/journal.pmen.0000196)
Supplement: S2 Table — Results of association testing including global model, selected model, and final model with bootstrap-derived quantities for multi-model inference and assessment of model uncertainty, male subgroup (n=165). (DOCX) [file pmen.0000196.s002.docx]

**S2 Table.** Results of association testing including global model, selected model, and final model with bootstrap-derived quantities for multi-model inference and assessment of model uncertainty, male subgroup (n=165).

|  | Global Model | | |  | Selected Model | | |  |  | Final Bootstrap Estimates | | | |
| --- | --- | --- | --- | --- | --- | --- | --- | --- | --- | --- | --- | --- | --- |
|  | $\hat{\beta_{g}}$ | 2.5th | 97.5th | Bootstrap inclusion frequency | $\hat{\beta_{s}}$ | 2.5th | 97.5th | RMSD Ratio | RC Bias | $\hat{\beta_{b}}$ | 2.5th | 97.5th | Std. $\hat{\beta_{b}}$ |
| (Intercept) | -6.619 | -22.690 | 9.451 | 100 | -2.027 | -22.690 | 9.451 | 1.314 | -38.526 | -2.249 | -38.199 | 4.647 | 0 |
| Age | 0.007 | -0.024 | 0.037 | 100 | 0.003 | -0.024 | 0.037 | 1.074 | -19.356 | 0.005 | -0.023 | 0.043 | 0.033 |
| BMI | 0.082 | -0.728 | 0.892 | 100 | -0.137 | -0.728 | 0.892 | 1.232 | -121.254 | -0.121 | -0.257 | 1.706 | -0.542 |
| ***CREBRF* rs373863828** | **-0.427** | **-0.753** | **-0.100** | **100** | **-0.441** | **-0.753** | **-0.100** | **0.894** | **-0.896** | **-0.419** | **-0.702** | **-0.134** | **-0.204** |
| **Abdominal circumference** | **0.086** | **0.041** | **0.130** | **98.1** | **0.081** | **0.041** | **0.130** | **1.215** | **-2.148** | **0.084** | **0.032** | **0.132** | **0.917** |
| Stress (PSS) | 0.058 | 0.004 | 0.113 | 79.3 | 0.053 | 0.004 | 0.113 | 1.273 | 14.008 | 0.056 | 0 | 0.116 | 0.226 |
| Social support (MSPSS) | 0.612 | 0.050 | 1.173 | 77.4 | 0.559 | 0.050 | 1.173 | 1.294 | 11.670 | 0.574 | 0 | 1.183 | 0.175 |
| Food security | 0.231 | -0.019 | 0.481 | 64.4 | 0.191 | -0.019 | 0.481 | 1.344 | 24.865 | 0.223 | 0 | 0.469 | 0.140 |
| SF-8 Mental health | 0.026 | -0.005 | 0.058 | 64.3 | 0.024 | -0.005 | 0.058 | 1.256 | 29.729 | 0.025 | 0 | 0.057 | 0.168 |
| Census region, NWU (Ref=AUA) | 0.113 | -0.524 | 0.750 | 59.0 |  |  |  | 0.767 | 65.608 | 0 | -0.297 | 0.721 | 0 |
| Census region, ROU (Ref=AUA) | 0.532 | -0.110 | 1.174 | 59.0 |  |  |  | 1.311 | 30.580 | 0.426 | 0 | 1.194 | 0.145 |
| SF-8 Physical health | 0.024 | -0.007 | 0.054 | 57.8 |  |  |  | 1.281 | 42.483 | 0.022 | 0 | 0.058 | 0.124 |
| Self-efficacy | -0.026 | -0.068 | 0.016 | 45.5 |  |  |  | 1.062 | 45.558 | 0 | -0.060 | 0 | 0 |
| Socioeconomic resources | -0.026 | -0.087 | 0.036 | 42.1 |  |  |  | 1.151 | 111.182 | 0 | -0.104 | 0.040 | 0 |
| Dietary pattern, Modern | 0.194 | -0.123 | 0.510 | 39.6 |  |  |  | 1.129 | 51.618 | 0 | 0 | 0.469 | 0 |
| Dietary pattern, Mixed traditional | -0.153 | -0.426 | 0.121 | 34.5 |  |  |  | 1.062 | 38.457 | 0 | -0.325 | 0 | 0 |
| Smoking, Yes (Ref=No) | -0.159 | -0.622 | 0.304 | 29.5 |  |  |  | 0.963 | 141.943 | 0 | -0.629 | 0 | 0 |
| Perceived social conflict | 0.002 | -0.042 | 0.047 | 24.6 |  |  |  | 0.891 | 136.957 | 0 | -0.044 | 0.048 | 0 |
| Alcohol, Yes (Ref=No) | -0.060 | -0.739 | 0.620 | 23.5 |  |  |  | 0.935 | -120.170 | 0 | -0.750 | 0.750 | 0 |
| Years of education | 0.008 | -0.076 | 0.093 | 23.0 |  |  |  | 0.900 | -40.876 | 0 | -0.080 | 0.099 | 0 |
| MVPA minutes/week, >0 (Ref=0) | 0.046 | -0.431 | 0.522 | 22.9 |  |  |  | 0.838 | 27.943 | 0 | -0.477 | 0.453 | 0 |
| Dietary pattern, Health conscious | -0.010 | -0.299 | 0.279 | 22.6 |  |  |  | 0.861 | -912.393 | 0 | -0.289 | 0.294 | 0 |
| Self-esteem | 0.006 | -0.053 | 0.065 | 15.9 |  |  |  | 0.723 | 149.728 | 0 | -0.056 | 0.057 | 0 |
| Fat mass index | -0.348 | -1.618 | 0.922 | 15.0 |  |  |  | 1.241 | 250.410 | 0 | -2.845 | 0 | 0 |
| Partnered, Yes (Ref=No) | -0.018 | -0.654 | 0.617 | 12.5 |  |  |  | 0.608 | 80.845 | 0 | -0.562 | 0.502 | 0 |
| Perceived discrimination | -0.011 | -0.071 | 0.049 | 11.6 |  |  |  | 0.628 | 104.393 | 0 | -0.056 | 0.036 | 0 |

Global and selected models presented alongside the final bootstrap model to assess the stability of the estimates and variable selection bias; global model shows the estimates and confidence intervals when all variables of interest are included in the model; selected model, selected via backward elimination with a significance level of 0.157 (AIC selection); final bootstrap estimates, indicate the bootstrap median estimate across all bootstrapped iterations; $\hat{\beta}$, unstandardized coefficient estimates with subscripts of g, s, and b corresponding to the global, selected, and bootstrapped models, respectively; 2.5th and 97.5th percentiles interpreted as limits of 95% confidence intervals; * symbol indicates variables included in all models based on *a priori* knowledge of the Samoan population or to adjust for study design strategy; estimated shrinkage factor of model 0.556; selected model frequency 0.6%; all variance inflation factors of selected model <2, with the exception of BMI and abdominal circumference, with variance inflation factors of 10.6 and 10.4, respectively; RMSD, root mean squared difference; global model, R^2^=29.7, adjusted R^2^=17.6; selected model, R^2^=25.6 adjusted R^2^=20.8; bolded values indicate statistical significance based on bootstrap confidence intervals.
