# Supplementary material for: Psychosocial correlates of HbA1c among adult Samoans without diabetes
Source: PLOS Ment Health. 2025 Feb 28;2(2):e0000196. doi: 10.1371/journal.pmen.0000196 (PMC12781966; doi:10.1371/journal.pmen.0000196)
Supplement: S3 Table — Results of association testing including global model, selected model, and final model with bootstrap-derived quantities for multi-model inference and assessment of model uncertainty, female subgroup (n=184). (DOCX) [file pmen.0000196.s003.docx]

**S3 Table.** Results of association testing including global model, selected model, and final model with bootstrap-derived quantities for multi-model inference and assessment of model uncertainty, female subgroup (n=184).

|  | Global Model | | |  | Selected Model | | |  |  | Final Bootstrap Estimates | | | |
| --- | --- | --- | --- | --- | --- | --- | --- | --- | --- | --- | --- | --- | --- |
|  | $\hat{\beta_{g}}$ | 2.5th | 97.5th | Bootstrap inclusion frequency | $\hat{\beta_{s}}$ | 2.5th | 97.5th | RMSD Ratio | RC Bias | $\hat{\beta_{b}}$ | 2.5th | 97.5th | Std. $\hat{\beta_{b}}$ |
| (Intercept) | -0.853 | -9.230 | 7.524 | 100 | 3.693 | -9.230 | 7.524 | 1.129 | -65.343 | 0.927 | -11.403 | 6.049 | 0 |
| **Age** | **0.045** | **0.013** | **0.078** | **100** | **0.037** | **0.013** | **0.078** | **1.085** | **-2.756** | **0.042** | **0.017** | **0.086** | **0.351** |
| BMI | 0.227 | -0.312 | 0.766 | 100 | 0.030 | -0.312 | 0.766 | 0.984 | -13.635 | 0.039 | -0.045 | 0.811 | 0.285 |
| ***CREBRF* rs373863828** | **-0.253** | **-0.498** | **-0.008** | **100** | **-0.249** | **-0.498** | **-0.008** | **0.792** | **-2.216** | **-0.244** | **-0.437** | **-0.067** | **-0.164** |
| Years of education | 0.094 | 0.001 | 0.188 | 75.1 | 0.081 | 0.001 | 0.188 | 1.355 | 20.676 | 0.088 | 0 | 0.218 | 0.167 |
| Dietary pattern, Mixed traditional | -0.176 | -0.370 | 0.019 | 71.3 | -0.169 | -0.370 | 0.019 | 1.356 | 34.302 | -0.178 | -0.423 | 0 | -0.142 |
| Smoking, Yes (Ref=No) | 0.303 | -0.076 | 0.682 | 60.9 | 0.319 | -0.076 | 0.682 | 1.331 | 42.554 | 0.290 | 0 | 0.751 | 0.124 |
| Self-efficacy | -0.024 | -0.063 | 0.015 | 54.0 | -0.024 | -0.063 | 0.015 | 1.035 | 47.879 | -0.021 | -0.060 | 0 | -0.107 |
| SF-8 Mental health | 0.007 | -0.015 | 0.028 | 45.1 |  |  |  | 1.317 | 72.010 | 0 | -0.023 | 0.034 | 0 |
| Fat mass index | -0.333 | -1.141 | 0.475 | 36.9 |  |  |  | 1.019 | 124.680 | 0 | -1.207 | 0 | 0 |
| Abdominal circumference | 0.010 | -0.017 | 0.038 | 36.6 |  |  |  | 1.062 | 121.956 | 0 | -0.014 | 0.040 | 0 |
| Census region, NWU (Ref=AUA) | 0.123 | -0.379 | 0.625 | 33.3 |  |  |  | 0.933 | 88.334 | 0 | -0.305 | 0.696 | 0 |
| Census region, ROU (Ref=AUA) | 0.146 | -0.335 | 0.627 | 33.3 |  |  |  | 0.834 | 106.531 | 0 | -0.058 | 0.583 | 0 |
| Partnered, Yes (Ref=No) | -0.108 | -0.574 | 0.357 | 32.6 |  |  |  | 1.185 | 180.463 | 0 | -0.795 | 0.371 | 0 |
| Social Support (MSPSS) | 0.154 | -0.223 | 0.530 | 31.9 |  |  |  | 0.878 | 108.928 | 0 | 0 | 0.449 | 0 |
| Dietary pattern, Health conscious | 0.030 | -0.138 | 0.198 | 28.1 |  |  |  | 0.943 | 207.329 | 0 | -0.143 | 0.209 | 0 |
| Stress (PSS) | 0.003 | -0.037 | 0.042 | 25.9 |  |  |  | 0.892 | 305.189 | 0 | -0.034 | 0.049 | 0 |
| Perceived social conflict | 0.005 | -0.029 | 0.038 | 24.3 |  |  |  | 0.884 | 286.060 | 0 | -0.024 | 0.042 | 0 |
| Food security | -0.043 | -0.169 | 0.084 | 22.6 |  |  |  | 0.843 | 107.857 | 0 | -0.142 | 0 | 0 |
| Self-esteem | 0.005 | -0.044 | 0.053 | 19.8 |  |  |  | 0.796 | 132.926 | 0 | -0.041 | 0.053 | 0 |
| MVPA minutes/week, >0 (Ref=0) | -0.064 | -0.540 | 0.411 | 17.6 |  |  |  | 0.638 | 313.361 | 0 | -0.442 | 0.005 | 0 |
| Perceived discrimination | -0.004 | -0.043 | 0.035 | 15.5 |  |  |  | 0.616 | 303.160 | 0 | -0.035 | 0.023 | 0 |
| Socioeconomic resources | 0.003 | -0.048 | 0.054 | 12.8 |  |  |  | 0.583 | 118.032 | 0 | -0.041 | 0.042 | 0 |
| SF-8 Physical health | -0.005 | -0.027 | 0.017 | 12.4 |  |  |  | 0.623 | 94.952 | 0 | -0.019 | 0.007 | 0 |
| Dietary pattern, Modern | -0.032 | -0.220 | 0.156 | 9.8 |  |  |  | 0.499 | 207.076 | 0 | -0.147 | 0 | 0 |

Global and selected models presented alongside the final bootstrap model to assess the stability of the estimates and variable selection bias; global model shows the estimates and confidence intervals when all variables of interest are included in the model; selected model, selected via backward elimination with a significance level of 0.157 (AIC selection); final bootstrap estimates, indicate the bootstrap median estimate across all bootstrapped iterations; $\hat{\beta}$, unstandardized coefficient estimates with subscripts of g, s, and b corresponding to the global, selected, and bootstrapped models, respectively; 2.5th and 97.5th interpreted as limits of 95% confidence intervals; * symbol indicates variables included in all models based on *a priori* knowledge of the Samoan population or to adjust for study design strategy; estimated shrinkage factor of model 0.689; selected model frequency 0.4%; all variance inflation factors of selected model <1.3; RMSD, root mean squared difference; global model, R^2^=20.4, adjusted R^2^=8.9; selected model, R^2^=16.9, adjusted R^2^=13.6; bolded values indicate statistical significance based on bootstrap confidence intervals.
