# Supplementary material for: Psychosocial correlates of HbA1c among adult Samoans without diabetes
Source: PLOS Ment Health. 2025 Feb 28;2(2):e0000196. doi: 10.1371/journal.pmen.0000196 (PMC12781966; doi:10.1371/journal.pmen.0000196)
Supplement: S1 Text — Abstract in Samoan. (DOCX) [file pmen.0000196.s004.docx]

**S1 Text: Translated Abstract**

**Mautinoa: Fa’amaopopoga fa’a-le-mafaufau ma aga-feso’ota’i mo le siaki’ina ole suka i tagata matutua i Samoa**

Ole malamalama i vaega fa’a-le-mafaufau fa’a-aga-feso’ota’i ole soifua maloloina i mea taua o su’esu’ega, maualuga le tulaga lamatia ole faitau aofa’i o tagata, lea e iai se a’afiaga tele o lea ituaiga malamalama’aga i taunuuga ole soifua maloloina. I Samoa, lea ua vave ona fa’ateleina ai le ta’atele ole ma’i suka ma ua silia ma le 30 tausaga, ma le feso’otaiga i mea e fai ile va ole mafaufau ma le ma’i suka ina ia tumau pea le malamalama’aga. Ole fa’amoemoe ole matou su’esu’ega ole fa’ailoaina lea o mea e feso’ota’i ma le siakiina ole ma’i suka i tagata matutua i Samoa i tusitusiga ma fa’amaumauga ole fa’atulagaina ole ma’i suka e aofia ai tausaga ole soifua, fa’atulagaga ole tino, tulaga lamatia tu’ufa’asolo o amioga. Tagata auai (n=349 tagata matutua) o ē o leā mauaina le tausiga o ma’i suka sa filifilia mai le su’esu’ega ole *Soifua Manuia* (2017-2019). Ole tele ole toe fo’i i tua ma le ave’esea ole pito i tua mai su’esu’ega ole mausalī ole fa’aaogaina e iloilo ai feso’otaiga i le va ole ma’i suka ma le faitau aofai o tagata auai, kenera (rs373863828 A allele in CREBRF), o mea moni, amioga ma aga-feso’otai (taua ole tagata lava ia, lagolago fa’a-aga-feso’otai, tulaga lelei e fa’atatau ile soifua maloloina ole olaga, fete’enaiga o feso’otaiga, ma le saogalemu o meaa’i). rs373863828 ole ituaiga tagata ma tausaga sa matua’i feso’otai lava ma kopi ole ma’i suka e sili atu ole rs373863828 A allele mo tausaga laiti sa feso’otai ma le maualalo ole ma’i suka. E ui e le’o ni vaega taua le fa’afuainumera e fa’avae i malologa o talitonuga, ae o i latou e sili atu le taua o le tagata lava ia ma le fa’atuatuaina ole tagata lava ia e foliga mai e maualalo, ae o i latou e maualuga atu le lagolago ma fa’afeso’otai ma e foliga e maualuga atu le ma’i suka. E fa’avae ile fa’aalia so’o o mea fa’a-le-mafaufau fa’a-aga-feso’ota’i i a tatou fa’ata’ita’iga, tatou te lagolagoina le fa’ateleina ole silafia ma le fa’alauiloaina ole soifua maloloina fa’a-le-mafaufau fa’a-aga-feso’otai i Samoa. Ole tu’ufaatasia ole soifua maloloina fa’a-le-mafaufau ma aga-feso’otai ma faiga masani fa’afoma’i ua maua ai se auala taua e fa’amalosia ai gasegase ole puipuiga ma le puleaina ole ma’i suka.
