# Supplementary material for: Psychosocial correlates of HbA1c among adult Samoans without diabetes
Source: PLOS Ment Health. 2025 Feb 28;2(2):e0000196. doi: 10.1371/journal.pmen.0000196 (PMC12781966; doi:10.1371/journal.pmen.0000196)
Supplement: S1 Code — Executable analysis code and a simulated data set. (DOCX) [file pmen.0000196.s005.docx]

**S1 Code.** To document full details of our analyses, we provide the executable analysis code and a simulated data set below.

# Acknowledgements

This code was adapted from the excellent work of Heinze, Wallisch, and Dunkler.

Heinze G, Wallisch C, Dunkler D. Variable selection - A review and recommendations for the practicing statistician. Biom J. 2018 May;60(3):431-449. doi: 10.1002/bimj.201700067. Epub 2018 Jan 2. PMID: 29292533; PMCID: PMC5969114.

# Load Libraries

library(tidyverse)

## ── Attaching packages ─────────────────────────────────────── tidyverse 1.3.1 ──

## ✔ ggplot2 3.4.4 ✔ purrr 1.0.2
## ✔ tibble 3.2.1 ✔ dplyr 1.1.2
## ✔ tidyr 1.1.4 ✔ stringr 1.5.0
## ✔ readr 2.1.0 ✔ forcats 0.5.1

## ── Conflicts ────────────────────────────────────────── tidyverse_conflicts() ──
## ✖ dplyr::filter() masks stats::filter()
## ✖ dplyr::lag() masks stats::lag()

library(shrink)

# Simulate data

# Set a random seed for reproducibility
set.seed(42)

# Number of observations
n <- 500

# Generate predictor variables
predictorA <- rnorm(n)
predictorB <- runif(n)
predictorC <- sample(0:1, n, replace = TRUE, prob = c(0.3, 0.7))
predictorD <- rnorm(n, mean = 5, sd = 2)
predictorE <- runif(n, min = 2, max = 7)
predictorF <- sample(0:1, n, replace = TRUE, prob = c(0.4, 0.6))
predictorG <- rnorm(n)
predictorH <- runif(n)

# Generate the outcome variable based on predictors
outcome <- 2 + 1.5 * predictorA + 0.8 * predictorB - 1.2 * predictorC + 0 * predictorD +
 0 * predictorE - 0.6 * predictorF + 2 * predictorG + 1.8 * predictorH + rnorm(n)

# Create a data frame with the simulated data
df <- data.frame(outcome, predictorA, predictorB, predictorC, predictorD, predictorE, predictorF)

# Convert factor variables
names <- c("predictorC", "predictorF")
df[, names] <- lapply(df[, names], factor)

# View the first few rows of the dataset
head(df)

## outcome predictorA predictorB predictorC predictorD predictorE predictorF
## 1 5.7588220 1.3709584 0.84829322 1 9.650117 3.368962 1
## 2 2.4543324 -0.5646982 0.06274633 0 6.048244 6.720984 1
## 3 0.3831973 0.3631284 0.81984509 1 6.941467 4.229917 1
## 4 1.5658075 0.6328626 0.53936029 0 5.753947 4.708936 0
## 5 -1.0487907 0.4042683 0.49902010 1 3.008133 2.808772 1
## 6 1.0240777 -0.1061245 0.02222732 1 3.805034 5.465268 1

# Perform variable selection

# EPV -------------------------------------------------------------- # Calculate number of events/variable
pred <- c("predictorA", "predictorB", "predictorC", "predictorD", "predictorE", "predictorF")
epv <- dim(df)[1]/length(pred)
epv

## [1] 83.33333

# Estimate full model ----------------------------------------------
formula <- paste("outcome~", paste(pred, collapse = "+"))
# Run model
full_mod <- lm(formula, data = df, x = T, y = T)
# Extract estimates
full_est <- coef(full_mod)
# Extract standard errors (SE)
full_se <- coef(summary(full_mod))[, "Std. Error"]
# Extract 95% confidence intervals (CI)
full_ci_low <- confint(full_mod)[, 1]
full_ci_high <- confint(full_mod)[, 2]
# Clean up
coef_names <- names(full_mod$coefficients)
# Summarize
summary(full_mod)

##
## Call:
## lm(formula = formula, data = df, x = T, y = T)
##
## Residuals:
## Min 1Q Median 3Q Max
## -6.5289 -1.5112 0.0166 1.4939 6.9133
##
## Coefficients:
## Estimate Std. Error t value Pr(>|t|)
## (Intercept) 2.75550 0.50085 5.502 6.05e-08 ***
## predictorA 1.45371 0.10299 14.116 < 2e-16 ***
## predictorB 0.90041 0.34755 2.591 0.009861 **
## predictorC1 -1.32366 0.21252 -6.229 1.01e-09 ***
## predictorD -0.02628 0.05187 -0.507 0.612673
## predictorE 0.07548 0.06841 1.103 0.270417
## predictorF1 -0.69612 0.20365 -3.418 0.000682 ***
## ---
## Signif. codes: 0 '***' 0.001 '**' 0.01 '*' 0.05 '.' 0.1 ' ' 1
##
## Residual standard error: 2.231 on 493 degrees of freedom
## Multiple R-squared: 0.3409, Adjusted R-squared: 0.3329
## F-statistic: 42.5 on 6 and 493 DF, p-value: < 2.2e-16

# Selected Model --------------------------------------------------- # Run model, forcing retainment of predictorA and predictorB into model # (i.e., don't subject predictors A or B to variable selection)
sel_mod <- step(lm(formula, data = df, x = T, y = T), direction = "backward", scope = list(upper = formula,
 lower = formula(outcome ~ predictorA + predictorB)), trace = 0)
# Summarize
summary(sel_mod)

##
## Call:
## lm(formula = outcome ~ predictorA + predictorB + predictorC +
## predictorF, data = df, x = T, y = T)
##
## Residuals:
## Min 1Q Median 3Q Max
## -6.5226 -1.5083 0.0549 1.4798 7.0820
##
## Coefficients:
## Estimate Std. Error t value Pr(>|t|)
## (Intercept) 3.0018 0.2639 11.373 < 2e-16 ***
## predictorA 1.4540 0.1028 14.142 < 2e-16 ***
## predictorB 0.8514 0.3451 2.467 0.01395 *
## predictorC1 -1.3391 0.2119 -6.320 5.84e-10 ***
## predictorF1 -0.6893 0.2030 -3.396 0.00074 ***
## ---
## Signif. codes: 0 '***' 0.001 '**' 0.01 '*' 0.05 '.' 0.1 ' ' 1
##
## Residual standard error: 2.23 on 495 degrees of freedom
## Multiple R-squared: 0.3388, Adjusted R-squared: 0.3335
## F-statistic: 63.42 on 4 and 495 DF, p-value: < 2.2e-16

# Extract estimates, SE, CI, etc.
sel_est <- sel_mod$coefficients[names(full_mod$coefficients)]
sel_se <- coef(summary(sel_mod))[, "Std. Error"][coef_names]
sel_ci <- confint(sel_mod)
sel_ci_named <- setNames(sel_ci[, 1], rownames(sel_ci))
sel_ci_low <- rep(NA, length(names(full_mod$coefficients)))
names(sel_ci_low) <- names(full_mod$coefficients)
sel_ci_low[names(sel_ci_named)] <- sel_ci_named
sel_ci_named <- setNames(sel_ci[, 2], rownames(sel_ci))
sel_ci_high <- rep(NA, length(names(full_mod$coefficients)))
names(sel_ci_high) <- names(full_mod$coefficients)
sel_ci_high[names(sel_ci_named)] <- sel_ci_named
sel_var <- grepl(paste(attr(terms(sel_mod), "term.labels"), collapse = "|"), pred) * 1
names(sel_var) <- pred

# Bootstrap -------------------------------------------------------- # Repeat backwards elimination across 1000 resamples of the data and extract # and store results
bootnum <- 1000
boot_est <- boot_se <- matrix(0, ncol = length(coef_names), nrow = bootnum, dimnames = list(NULL,
 coef_names))
boot_var <- matrix(0, ncol = length(pred), nrow = bootnum, dimnames = list(NULL,
 pred))

set.seed(5437854)
for (i in 1:bootnum) {
 data_id <- sample(1:dim(df)[1], replace = T)
 boot_mod <- step(lm(formula, data = df[data_id, ], x = T, y = T), scope = list(upper = formula,
 lower = formula(outcome ~ predictorA + predictorB)), direction = "backward",
 trace = 0)
 boot_est[i, names(coef(boot_mod))] <- coef(boot_mod)
 boot_se[i, names(coef(boot_mod))] <- coef(summary(boot_mod))[, "Std. Error"]
 boot_var[i, attr(terms(boot_mod), "term.labels")] <- 1
}
# Calculate bootstrap inclusion frequency
boot_01 <- (boot_est != 0) * 1
boot_inclusion <- apply(boot_01, 2, function(x) sum(x)/length(x) * 100)

# Overview of estimates and measures -------------------------------- # Calculate model stability and summarize results
sqe <- (t(boot_est) - full_est)^2
rmsd <- apply(sqe, 1, function(x) sqrt(mean(x)))
rmsdratio <- rmsd/full_se
boot_mean <- apply(boot_est, 2, mean)
boot_meanratio <- boot_mean/full_est
boot_relbias <- (boot_meanratio/(boot_inclusion/100) - 1) * 100
boot_median <- apply(boot_est, 2, median)
boot_025per <- apply(boot_est, 2, function(x) quantile(x, 0.025))
boot_975per <- apply(boot_est, 2, function(x) quantile(x, 0.975))
overview <- round(cbind(full_est, full_se, full_ci_low, full_ci_high, boot_inclusion,
 sel_est, sel_se, sel_ci_low, sel_ci_high, rmsdratio, boot_relbias, boot_median,
 boot_025per, boot_975per), 4)
overview <- overview[order(overview[, "boot_inclusion"], decreasing = T), ]
overview

## full_est full_se full_ci_low full_ci_high boot_inclusion sel_est
## (Intercept) 2.7555 0.5008 1.7714 3.7396 100.0 3.0018
## predictorA 1.4537 0.1030 1.2514 1.6561 100.0 1.4540
## predictorB 0.9004 0.3476 0.2175 1.5833 100.0 0.8514
## predictorC1 -1.3237 0.2125 -1.7412 -0.9061 100.0 -1.3391
## predictorF1 -0.6961 0.2036 -1.0962 -0.2960 98.6 -0.6893
## predictorE 0.0755 0.0684 -0.0589 0.2099 39.0 NA
## predictorD -0.0263 0.0519 -0.1282 0.0756 22.4 NA
## sel_se sel_ci_low sel_ci_high rmsdratio boot_relbias boot_median
## (Intercept) 0.2639 2.4832 3.5204 1.0280 1.4140 2.8397
## predictorA 0.1028 1.2520 1.6560 1.0075 0.0941 1.4534
## predictorB 0.3451 0.1734 1.5295 0.9483 -1.2145 0.8802
## predictorC1 0.2119 -1.7554 -0.9228 0.9718 0.3883 -1.3169
## predictorF1 0.2030 -1.0882 -0.2905 0.9784 -0.2134 -0.6921
## predictorE NA NA NA 1.1303 92.3454 0.0000
## predictorD NA NA NA 0.9354 174.8319 0.0000
## boot_025per boot_975per
## (Intercept) 1.7749 3.7304
## predictorA 1.2648 1.6737
## predictorB 0.2434 1.5254
## predictorC1 -1.7301 -0.9497
## predictorF1 -1.0480 -0.3154
## predictorE 0.0000 0.2111
## predictorD -0.1322 0.0785

# Model frequency --------------------------------------------------- # Calculate additional measures of model stability
boot_var_count <- cbind(boot_var, count = rep(1, times = bootnum))
boot_modfreq <- aggregate(count ~ ., data = boot_var_count, sum)
boot_modfreq[, "percent"] <- boot_modfreq$count/bootnum * 100
boot_modfreq <- boot_modfreq[order(boot_modfreq[, "percent"], decreasing = T), ]
boot_modfreq[, "cum_percent"] <- cumsum(boot_modfreq$percent)
boot_modfreq <- boot_modfreq[boot_modfreq[, "cum_percent"] <= 80, ]
if (dim(boot_modfreq)[1] > 20) {
 boot_modfreq <- boot_modfreq[1:20, ]
}

num_rows <- nrow(boot_modfreq)
mods <- data.frame(Predictors = apply(boot_modfreq[, -num_rows], 1, function(x) paste(names(x[x ==
 1]), collapse = " ")), count = boot_modfreq$count, percent = boot_modfreq$percent,
 cum_percent = boot_modfreq$cum_percent)
rownames(mods) <- seq_len(nrow(mods))

# Model frequency in % of selected model ----------------------------
sel_modfreq <- sum(apply(boot_var, 1, function(x) identical(sel_var, x)))/bootnum *
 100
sel_modfreq

## [1] 46.1

# Pairwise inclusion frequency in % ----------------------------------
pval <- 0.01
boot_pairfreq <- matrix(100, ncol = length(pred), nrow = length(pred), dimnames = list(pred,
 pred))

expect_pairfreq <- NULL
combis <- combn(pred, 2)

for (i in 1:dim(combis)[2]) {
 boot_pairfreq[combis[1, i], combis[2, i]] <- sum(apply(boot_var[, combis[, i]],
 1, sum) == 2)/bootnum * 100
 expect_pairfreq[i] <- boot_inclusion[grepl(combis[1, i], names(boot_inclusion))][1] *
 boot_inclusion[grepl(combis[2, i], names(boot_inclusion))][1]/100
 boot_pairfreq[combis[2, i], combis[1, i]] <- ifelse(is(suppressWarnings(try(chisq.test(boot_var[,
 combis[1, i]], boot_var[, combis[2, i]]), silent = T)), "try-error"), NA,
 ifelse(suppressWarnings(chisq.test(boot_var[, combis[1, i]], boot_var[, combis[2,
 i]])$p.value) > pval, "", ifelse(as.numeric(boot_pairfreq[combis[1, i],
 combis[2, i]]) < expect_pairfreq[i], "-", "+")))
}
diag(boot_pairfreq) <- apply(boot_var, 2, function(x) sum(x)/length(x) * 100)
print(boot_pairfreq, quote = F)

## predictorA predictorB predictorC predictorD predictorE predictorF
## predictorA 100 100 100 22.4 39 98.6
## predictorB <NA> 100 100 22.4 39 98.6
## predictorC <NA> <NA> 100 22.4 39 98.6
## predictorD <NA> <NA> <NA> 22.4 8.2 22
## predictorE <NA> <NA> <NA> 39 38.6
## predictorF <NA> <NA> <NA> 98.6

# Shrinkage factors --------------------------------------------------
sel_mod_shrinkg <- shrink(sel_mod, type = "global")
sel_mod_shrinkp <- shrink(sel_mod, type = "parameterwise")

sel_mod_shrinkg$ShrinkageFactors

## [1] 0.9828539

sel_mod_shrinkp$ShrinkageFactors

## predictorA predictorB predictorC1 predictorF1
## 0.9936462 0.8449801 0.9722897 0.9103587

sel_mod_shrinkp_vcov <- vcov(sel_mod_shrinkp)

## (Intercept) predictorA predictorB predictorC1 predictorF1
## (Intercept) 0.070768200 3.89118e-04 -6.73710e-02 0.02126890 0.033612600
## predictorA 0.000389118 5.07029e-03 7.34269e-05 0.00049074 -0.000588754
## predictorB -0.067371000 7.34269e-05 1.66613e-01 0.00158518 0.001180560
## predictorC1 0.021268900 4.90740e-04 1.58518e-03 0.02543140 -0.001902400
## predictorF1 0.033612600 -5.88754e-04 1.18056e-03 -0.00190240 0.088087700

round(cbind(`Shrinkage factors` = sel_mod_shrinkp$ShrinkageFactors, `SE of shrinkage factors` = sqrt(diag(sel_mod_shrinkp_vcov))[-1],
 `Correlation matrix of shrinkage factors` = cov2cor(sel_mod_shrinkp_vcov)[-1,
 -1]), 4)

## Shrinkage factors SE of shrinkage factors predictorA predictorB
## predictorA 0.9936 0.0712 1.0000 0.0025
## predictorB 0.8450 0.4082 0.0025 1.0000
## predictorC1 0.9723 0.1595 0.0432 0.0244
## predictorF1 0.9104 0.2968 -0.0279 0.0097
## predictorC1 predictorF1
## predictorA 0.0432 -0.0279
## predictorB 0.0244 0.0097
## predictorC1 1.0000 -0.0402
## predictorF1 -0.0402 1.0000

# Session Information

sessionInfo()

## R version 4.1.2 (2021-11-01)
## Platform: x86_64-pc-linux-gnu (64-bit)
## Running under: CentOS Linux 7 (Core)
##
## Matrix products: default
## BLAS: /usr/local/lib64/R/lib/libRblas.so
## LAPACK: /usr/local/lib64/R/lib/libRlapack.so
##
## locale:
## [1] LC_CTYPE=en_US.UTF-8 LC_NUMERIC=C
## [3] LC_TIME=en_US.UTF-8 LC_COLLATE=en_US.UTF-8
## [5] LC_MONETARY=en_US.UTF-8 LC_MESSAGES=en_US.UTF-8
## [7] LC_PAPER=en_US.UTF-8 LC_NAME=C
## [9] LC_ADDRESS=C LC_TELEPHONE=C
## [11] LC_MEASUREMENT=en_US.UTF-8 LC_IDENTIFICATION=C
##
## attached base packages:
## [1] stats graphics grDevices utils datasets methods base
##
## other attached packages:
## [1] shrink_1.2.2 forcats_0.5.1 stringr_1.5.0 dplyr_1.1.2
## [5] purrr_1.0.2 readr_2.1.0 tidyr_1.1.4 tibble_3.2.1
## [9] ggplot2_3.4.4 tidyverse_1.3.1 knitr_1.44
##
## loaded via a namespace (and not attached):
## [1] TH.data_1.1-0 colorspace_2.1-0 ellipsis_0.3.2
## [4] class_7.3-19 htmlTable_2.3.0 base64enc_0.1-3
## [7] fs_1.6.3 rstudioapi_0.13 listenv_0.8.0
## [10] MatrixModels_0.5-0 mvtnorm_1.1-3 prodlim_2019.11.13
## [13] fansi_1.0.5 lubridate_1.9.2 xml2_1.3.5
## [16] codetools_0.2-18 splines_4.1.2 Formula_1.2-4
## [19] jsonlite_1.8.7 pROC_1.18.0 caret_6.0-90
## [22] broom_0.7.10 cluster_2.1.2 dbplyr_2.1.1
## [25] png_0.1-7 compiler_4.1.2 httr_1.4.2
## [28] backports_1.3.0 assertthat_0.2.1 Matrix_1.3-4
## [31] fastmap_1.1.1 cli_3.6.1 formatR_1.11
## [34] htmltools_0.5.6.1 quantreg_5.86 tools_4.1.2
## [37] gtable_0.3.4 glue_1.6.2 reshape2_1.4.4
## [40] Rcpp_1.0.11 cellranger_1.1.0 vctrs_0.6.4
## [43] nlme_3.1-153 conquer_1.2.1 iterators_1.0.14
## [46] timeDate_3043.102 xfun_0.40 gower_0.2.2
## [49] globals_0.14.0 rvest_1.0.2 timechange_0.2.0
## [52] lifecycle_1.0.3 polspline_1.1.19 future_1.23.0
## [55] zoo_1.8-12 MASS_7.3-60 scales_1.2.1
## [58] ipred_0.9-12 hms_1.1.1 sandwich_3.0-1
## [61] parallel_4.1.2 SparseM_1.81 RColorBrewer_1.1-3
## [64] mfp_1.5.2.2 yaml_2.3.7 gridExtra_2.3
## [67] rms_6.2-0 rpart_4.1-15 latticeExtra_0.6-29
## [70] stringi_1.7.12 foreach_1.5.2 checkmate_2.1.0
## [73] hardhat_0.2.0 lava_1.6.10 matrixStats_0.61.0
## [76] rlang_1.1.1 pkgconfig_2.0.3 evaluate_0.22
## [79] lattice_0.20-45 recipes_0.2.0 htmlwidgets_1.6.2
## [82] tidyselect_1.2.0 parallelly_1.28.1 plyr_1.8.8
## [85] magrittr_2.0.3 R6_2.5.1 generics_0.1.3
## [88] Hmisc_4.6-0 multcomp_1.4-17 DBI_1.1.1
## [91] pillar_1.9.0 haven_2.4.3 foreign_0.8-81
## [94] withr_2.5.1 survival_3.2-13 nnet_7.3-16
## [97] future.apply_1.8.1 modelr_0.1.8 crayon_1.5.2
## [100] utf8_1.2.3 tzdb_0.2.0 rmarkdown_2.25
## [103] jpeg_0.1-9 grid_4.1.2 readxl_1.3.1
## [106] data.table_1.14.2 ModelMetrics_1.2.2.2 reprex_2.0.1
## [109] digest_0.6.33 stats4_4.1.2 munsell_0.5.0
